# Selected Formulæ

**Published:** 1899-09

**Authors:** 


### Selected Formulæ.

---

**Post-Partum Hemorrhage.**—

℞ Ergotine.............................. ʒj

Chloral ................................ ʒss

Aq. dest............................q. s. ad℥j

M. Sig. Ten or twenty minims injected deeply into the muscles of the buttock.—*Simpson, Med. Rec.*

---

**Later Stages of Gonorrhoea.**—

℞ Terebinth. alb........................ ʒj

Res. Podoph...........................gr. ss

Camphor monobrom.................... ʒj

M. et ft. pil. No. xxx. Sig. One pill four times a day.—*The Medical Bulletin.*

---

**Soft Corns.**—

℞ Iodine.................................... gr.j

Flexible collodion.........................fl.ʒiij

Alcohol .................................. fl.ʒj

Potassium iodide....................... gr.ij

M. Sig. Paint the corn every night.—*Pacific Rec. of Med. and Surg.*

---

**Blackheads.**—

℞ Ichthyol,

Bismuth subnitr.,

Ammoniat. mercury........................ ʒaaj

Vaseline .................................. ʒx

M. ft. ungt. Sig. Apply at night.—*Louisville Medical Monthly.*

**Acute Tonsillitis.**—Dr. George Fay, in the *Atlanta Medical and Surgical Journal*, recommends the following:

℞ Tinct. Aconite................................ʒss

Chloroform-water......... ..................℥ii

Distilled water..............................ʒiv

A teaspoonful every five minutes for twelve doses; afterwards a dose every hour. If necessary repeat the mixture and direct the repetition to be taken as before, beginning with five-minute doses.

---

**Infantile Diarrhoea.**—A writer in the *Med. Sum.* gives the following formula as one he has used with great success for the past twenty years in cases of infantile diarrhœa:

℞ Syr. rhi aromatic,

Mucil. acacia........................ aa ℥j

Bismuth subnitrate................. ʒj

Spr. ammon. aromat.,

Syr. ipecas ...........................aa gtt. xx

M. Sig. A teaspoonful every three hours to a child one year old.—*Exchange.*

---

**Neurasthenic Headaches.**—Dr. Joseph Collins states that in neurasthenic headaches, associated with low vascular tension, caffein, either alone or in combination, gives excellent results. The following formula he has found particularly useful:

℞ Caffeine citrate....................... 5 grains
Sodium bromid.........................10 grains
Sodium bicarbonate.................10 grains
Pulv. tartaric acid...................10 grains

M. Make into one powder.
Sig. Take in water while effervescing.

Or—

℞ Caffeine salicylate .....................1 grain
Ammonium salicylate } of each...5 grains
Phenol salicylate }

M. Make one capsule.
Sig. One capsule every three to four hours.

Or—

℞ Caffein..........................½ to 1½ grains

Phenacetin .................... 5 grains

M. Make one capsule.
Sig. Take with hot water, and repeat in one hour.

